# Health system constraints in cervical cancer prevention in rural Namibia: A qualitative study

**DOI:** 10.4102/phcfm.v17i1.4976

**Published:** 2025-08-21

**Authors:** Elizabeth K. Ndakukamo, Roshwitha Mahalie, Panduleni Hailonga-van Dijk

**Affiliations:** 1 Department of Preventative Health Sciences, Faculty of Health, Natural Resources and Applied Sciences, Namibia University of Science and Technology (NUST), Windhoek, Namibia; 2 Seahorse Research and Training Institute (SERTI), Windhoek, Namibia

**Keywords:** cervical, cancer, prevention, health system, challenges, health infrastructure, Namibia

## Abstract

**Background:**

Cervical cancer remains a pressing public health concern in Namibia, with significant barriers to prevention, particularly in rural areas.

**Aim:**

This study explored health system’s challenges and their impact on cervical cancer prevention efforts.

**Setting:**

This study was conducted in the Ohangwena and Kavango West regions of Namibia.

**Methods:**

A qualitative exploratory design was employed, focusing on healthcare workers directly involved in cervical cancer prevention. In-depth interviews were utilised to collect data from 11 participants from four district hospitals. Thematic analysis, guided by the World Health Organisation’s six health system framework pillars, was used.

**Results:**

Key service gaps were identified across critical areas of cervical cancer prevention, including a lack of awareness, a lack of human papillomavirus vaccines and referral screening equipment that limited local outreach services. Other significant findings included the shortage of trained personnel and the manual data systems, which resulted in deficiencies in decision-making. Financial constraints, including reliance on non-governmental organisation funding and weak community engagement, exacerbated by cultural stigma, presented leadership challenges.

**Conclusion:**

Investing in human resources for health, decentralising budget trends and enhancing data systems are critical for cervical cancer prevention in rural Namibia. Equally important is the active involvement of the community in these efforts.

**Contribution:**

This study highlights key health system constraints in the Ohangwena and Kavango West regions.

## Introduction

Cervical cancer continues to be a significant public health concern, with a pronounced variation in its incidence and mortality rates globally.^1^ In the developed world, awareness of cervical cancer is significantly higher compared to the developing world.^2^ In sub-Saharan Africa (SSA), approximately a third of the general population knows about cervical cancer.^3^ In 2022, there were 662 301 newly diagnosed cases of cervical cancer and 348 874 fatalities worldwide, with over 94% of these deaths occurring in developing countries.^4^ Cervical cancer is the leading cause of cancer-related deaths among women in Africa, having a mortality rate of 23.1%.^5^ SSA experiences a significant impact, with 40 new cases diagnosed per 100 000 women each year.^4^ Though recent national statistics on cervical cancer incidence are limited in Namibia, the Global Cancer Observatory (GLOBOCAN) estimates that cervical cancer ranks as the second most prevalent after breast cancer in Namibia, with approximately 350 new cases and 203 deaths reported in 2022.^4^ The leading cause of cervical cancer is a long-lasting infection with the human papillomavirus (HPV).^6^

Over the years, healthcare systems in Africa have suffered from numerous challenges that have cut across institutional, financial, human resources, technical and political development.^7^ In light of this, the World Health Organization (WHO) developed a framework in 2007 that breaks down healthcare systems into six fundamental pillars, or ‘building blocks’, that is service delivery (provision of healthcare services), healthcare workforce (availability and distribution of healthcare workers), healthcare information systems (management of health information), medicinal products, vaccines and technology (access to essential medicines and technologies), financing (funding of healthcare services) and leadership and governance (management and oversight of healthcare systems).^8^ The bulk of African countries lack the fundamental infrastructure needed for effective healthcare systems.^7,9,10^

Namibia faces challenges in the healthcare delivery sector that have influenced the high prevalence and incidence of cervical cancer. These challenges include a lack of awareness, a lack of HPV vaccines in the public health sector, which is the primary prevention method for cervical cancer; and a limited healthcare service delivery infrastructure.^3^ Other prevalent healthcare challenges include a significant shortage of specialised healthcare professionals, mainly because of limited local training opportunities.^9^ As part of efforts to remedy the shortage of specialised healthcare professionals, aspiring medical specialists have frequently been sent to South African universities for advanced training, resulting in high costs and extended vacancies in essential healthcare roles.^9^ Furthermore, indirect financial obstacles worsen the situation because many women in rural areas cannot cover the costs related to transportation to healthcare facilities for screening and treatment, as over 40% of Namibians do not live within a five-kilometre radius, as recommended by WHO.^10^ Additionally, the lack of solid policy backing and erratic acquisition of medical products and technologies cause disruptions in service delivery.^9^ At the same time, inadequate health data management hinders effective follow-up and continuity of care.^11^

Several studies have documented that cervical cancer prevention (CECAP) programmes are present in the healthcare systems of most developing nations.^12,13,14,15^ Nevertheless, health systems with screening and treatment programmes encounter challenges, resulting in cervical cancer screening coverage that is significantly lower than the WHO-recommended 70% of the targeted population during the first 10 years of implementation.^16^ Maseko et al. argue that inadequate staff training in screening, treatment, referral and record keeping is some of the health system challenges that have contributed to the failure of health systems in implementing effective CECAP programmes.^15^ In addition, they found that there was poor management of cervical cancer at the district level, coupled with inadequate service providers and a lack of essential equipment.^15^ These findings were similar to those of Ebu et al.,^17^ who found that the lack of healthcare workers and the availability of screening and treatment centres located mainly in urban areas were the most challenging. These challenges collectively undermine the effectiveness of CECAP programmes across developing countries.^2^

There is a paucity of studies on CECAP, screening and treatment in Namibia. Though multiple studies have addressed prostate cancer^18,19,20^ and breast cancer,^21,22^ only two studies examined the issues related to cervical cancer screening at regional health facilities.^23,24^ No studies across the country have investigated the challenges encountered by the Namibian healthcare system in detecting cervical cancer incidence. So far, insufficient focus has been given to tackling the specific challenges encountered by the healthcare system in rural areas, especially at the community-based level. Arguably, all the foregoing may impede effective CECAP and control in Namibia’s Ohangwena and Kavango West regions.

## Research methods and design

### Study design and setting

The study employed an exploratory qualitative design to explore the health system challenges in CECAP. The study was conducted in the Ohangwena and Kavango West regions, two predominantly rural regions located in northern Namibia. Ohangwena is a densely populated region bordering Angola, with a population of 261 691 people. Approximately, 94% of this population resides in rural areas.^25^ The Kavango West region also borders Angola, with a population of 123 266; like Ohangwena, a significant number of inhabitants reside in rural areas.^25^ Both regions have well-documented challenges in healthcare services and service delivery, and they were selected because of their high prevalence of cervical cancer, inadequate prevention and screening services, and known disparities in health infrastructure, which may contribute to poor prevention efforts. The specific study locations included three health districts in the Ohangwena region (Okongo, Eenhana and Engela) and the only health district in the Kavango West region (Nankudu).

### Study population and sampling strategy

The target population was healthcare workers (nurses, doctors and community health extension workers) involved in CECAP. This study employed a purposive sampling method to identify healthcare workers with firsthand experience of CECAP across the Ohangwena and Kavango West regions. Eleven participants were recruited from all the district hospitals in both regions. In addition, participants were approached through formal invitations sent through the office of their regional health directors. Healthcare workers from the two regions with at least a year of working experience in CECAP services were included in the study. This ensured insights from experienced individuals directly involved in cervical cancer management. Staff with less than one year in their role, those not directly involved in CECAP and those from outside the targeted health districts were excluded from the study.

### Data collection

An in-depth interview guide was used to conduct face-to-face interviews in English from June to August 2024. The guide’s themes were guided by the pillars of the WHO health system frameworks.^8^ The instrument was divided into seven sections. Section one gathered socio-demographic details about participants, while the remaining sections examined the health system challenges based on the six pillars as foundational elements of a health system: health services delivery, workforce, health information, leadership and governance, essential medical products, vaccines and technologies, and financing. Participants were given time to review the information sheet, and the researcher additionally clarified the aim of the study, detailing the anticipated length of the interview and the use of the audio recorder. Written informed consent was obtained before the interviews were conducted. The interviews took place in a private room within the hospital to ensure confidentiality at times that were convenient for the participants. Each interview lasted between 26 and 48 min and was recorded using a mobile phone. Data saturation occurred after interviewing nine participants. However, the researchers interviewed two more participants to declare data saturation.

### Data analysis

The socio-demographic data were analysed descriptively in Microsoft Excel and presented in a frequency table. The interviews were transcribed verbatim 24 h after each interview. An inductive thematic analysis approach was conducted, guided by the Braun and Clarke Framework.^26^ The transcripts were coded to identify critical insights related to health system constraints in CECAP using ATLAS. Ti 23 software. A codebook was created in Microsoft Word to integrate and collate the codes, after which overlapping themes and subthemes were identified. To ensure trustworthiness and rigour, the guide was piloted with two healthcare workers from the Kavango West region who were not part of the main study to assess the relevance and clarity of the questions. Additionally, literature control was performed to compare the results with those of existing literature. Two independent coders reviewed and analysed the transcripts to ensure reliability.

### Ethical considerations

Ethical clearance to conduct this study was obtained from the Research Ethics Committee of the Faculty of Health, Natural Resources and Applied Sciences at the Namibia University of Science and Technology (NUST) (No. FHNRAS:58/2023) and the Ministry of Health and Social Services Ethics Committee (No. 22/3/1/2). Permission to conduct the study in the selected settings was granted by the health regional directors for each region. Following the complete presentation of the study’s objectives and the expectations surrounding their participation, all participants gave their written consent. Participation was entirely voluntary, and transcripts were anonymised at reporting to protect the participants’ privacy. The data were stored and password protected on the personal computer of the corresponding author without access to any third parties to ensure confidentiality.

## Results

### Socio-demographic characteristics of participants

Eleven participants agreed to participate in the study; seven were females, and four were males. The demographic characteristics of the participants are outlined in Table 1.

**TABLE 1:** Socio-demographic characteristics of participants (*N* = 11).

Variables	Categories	*n*	%
Age (years)	18–29	1	9.0
	30–35	4	36.4
	> 35	6	54.6
Gender	Female	7	63.6
	Male	4	36.4
Education level	Diploma	2	18.2
	Bachelor’s degree	7	63.6
	Master’s degree	2	18.2
Occupation	Doctors	4	36.4
	Nurses	6	54.5
	Health extension worker	1	9.0
Working experience (years)	1–5	4	36.3
	6–10	3	27.3
	> 10	4	36.4
Region	Kavango West	6	54.6
	Ohangwena	5	45.4
Employer	Government	6	54.5
	NGO	5	45.5

NGO, non-governmental organisation.

### Emerging themes

The findings are structured into six main themes: service delivery, health workforce, health information, health financing, essential medical products and technology and leadership and governance. The outline of themes, sub-themes and key findings is presented in Table 2.

**TABLE 2:** Outline of themes, sub-themes and key findings.

Theme	Sub-theme	Key findings
1. Service delivery	1.1. Cervical cancer screening services	Clinics offer VIA and Pap smear screenings, but service interruptions occur because of shortages and logistical constraints, particularly in rural areas.
	1.2. HPV vaccine accessibility	HPV vaccines are primarily available in the private sector, with limited access to public health facilities.
	1.3. Community outreach services	Outreach efforts are mainly clinic based, with occasional campaigns coordinated with community leaders. Resource and transportation challenges affect coverage.
2. Health workforce	2.1. Staff shortages and retention	High turnover rates among healthcare workers, especially young professionals. Rural postings and lack of incentives contribute to retention challenges. Heavy reliance on NGO-funded positions raises sustainability concerns.
	2.2. Training and specialisation	Few healthcare workers receive specialised training in cervical cancer screening. There is a shortage of gynaecologists and oncologists.
3. Health information	3.1. Data collection and management	Data collection is mostly manual, with limited use of electronic systems. Some districts have basic digital systems, but connectivity and equipment limitations pose challenges.
4. Health financing	4.1. Budget constraints	No dedicated government budget for cervical cancer prevention. Services rely heavily on NGO funding, raising concerns about long-term sustainability.
5. Access to essential medicines	5.1. Supplies for screening services	Frequent shortages of VIA and Pap smear kits disrupt service delivery. HPV vaccines are largely unavailable in public facilities.
	5.2. Equipment and infrastructure	Screening equipment is outdated, and storage facilities are inadequate. Basic infrastructure, such as examination beds, needs improvement to meet patient needs.
6. Leadership and governance	6.1. Prevention policy	Leadership oversight is present, but strategic support is insufficient. Governance relies heavily on NGO funding because of the lack of a dedicated cervical cancer policy.
	6.2. Community involvement	Collaboration with community leaders is inconsistent. There is a lack of local advocates for cervical cancer awareness, and cultural stigma remains a barrier to screening.

VIA, visual inspection with acetic acid; HPV, human papillomavirus; NGO, non-governmental organization.

### Theme 1: Service delivery

High-quality service delivery ensures that personal and non-personal health interventions are safe, effective and accessible to those in need at the right time and place.^8^ This theme highlights the inadequate access and inconsistency of screening, vaccination and community outreach services.

#### Sub-theme 1.1: Cervical cancer screening services

Cervical cancer screening services, including Visual Inspection with Acetic acid (VIA) and Pap smear tests, are available in all the district hospitals and some clinics. However, these services often face interruptions because of transport challenges and shortages of staff. A participant noted:

‘There are times we have to cancel trips to clinics because there is no car, even if the booking was made way earlier.’ (P1, female, 28 years old)

‘I am the only CECAP nurse here, and if I go on leave, then there is no one to screen, so patients should wait for me to come back.’ (P4, female, 48 years old)

This shortage hinders continuous access to cervical cancer screening services. While coverage was generally positive, consistency was affected by some challenges. Resource constraints occasionally slowed service provisions at the clinic, as noted by one participant:

‘It’s supposed to be in all clinics, but sometimes, due to staff and equipment shortage, you find some of the clinics stopped somewhere.’ (P6, female, 32 years old)

#### Sub-theme 1.2: HPV vaccine accessibility

About 80% of the participants identified the availability of HPV vaccines as a critical issue, as these vaccines are primarily accessible through private-sector services only. The Namibian public health facilities have not introduced the HPV vaccine to date, and the participants were not even aware of any rollout, leaving lower-income populations underserved. One respondent stated:

‘Unfortunately, we cannot offer HPV to everyone who needs it, especially the patients who use public health services. It does not exist, but if you have medical aid, I am not sure, but I think you can get it in private.’ (P3, male, 51 years old)

#### Sub-theme 1.3: Community outreach services

Participants discussed varying views on outreach services, with some acknowledging the community sensitisation efforts through health talks on the community radio channel and the involvement of traditional leaders and Community Health workers. However, these efforts were often obstructed by logistical challenges. For instance, one participant indicated that:

‘Community campaigns are coordinated with traditional leaders, but screening primarily occurs in clinic settings, not really in the community, due to bad roads and long distances. Every clinic is given a specific date for when we are coming.’ (P1, female, 28 years old)

### Theme 2: Health workforce

A responsive, fair and efficient health workforce works to achieve the best possible health outcomes, optimising available resources and adapting to prevailing circumstances.^8^ The health workforce theme reflects emerging constraints related to staffing shortages, struggles with staff retention and insufficient specialised training in CECAP.

#### Sub-theme 2.1: Staff shortages and retention

Approximately 90% of the participants cited that the availability of trained healthcare staff was a prominent challenge, with many clinics served by only one or two CECAP-trained nurses and doctors, which limits service continuity and outreach capacity. Staff turnover was an ongoing issue, particularly in remote areas, where retention was almost impossible. One participant shared:

‘They come here to get into the system, then they want to leave. Housing and bush allowances are provided, but many nurses still transfer out to cities.’ (P3, male, 51 years old)

‘It’s hard to keep young professionals in these rural postings; they leave for better opportunities elsewhere.’ (P9, male, 40 years old)

Some participants also discussed the reliance on non-government organization (NGO)-funded positions, which further raises concerns about the sustainability of staffing:

‘All of us are conducting this service under NGO support. One day, when the NGO pulls out, there will be a huge challenge.’ (P7, female, 39 years old)

#### Sub-theme 2.2: Training and specialisation

Most participants stressed the lack of specialised staff in CECAP and treatment, including trained gynaecologists and oncologists. Ohangwena region had one gynaecologist and Kavango west region had none. Most healthcare workers have minimal training in cervical cancer screening techniques, affecting the quality and reach of services. A respondent remarked:

‘We don’t have enough staff with specialised training, which limits the services we can provide.’ (P5, male, 37 years old)

‘If the patient comes in with an advanced stage of cervical cancer, we refer them to the Intermediate Hospital Oshakati (IHO). There is no capacity here.’ (P8, female, 36 years old)

### Theme 3: Health information

A strong health information system allows the production, analysis, dissemination and use of accurate and timely data on health determinants, health system performance and population health status.^8^ This theme focuses on the challenges in data collection and confidentiality that impact data-driven decisions in CECAP.

#### Sub-theme 3.1: Data collection and management

Participants from both regions reported that data collection largely relies on manual processes, with only a few districts employing basic digital systems. However, Internet connectivity issues and basic equipment shortages, such as computers, hamper digital data collection and management efforts. Some staff members noted:

‘Our data collection is still mostly manual, and without proper connectivity, digital options are hard to implement.’ (P1, female, 28 years old)

‘We don’t have enough computers because you would want them at each point of the care. The CDC should have, but most departments do not have.’ (P2, female, 35 years old)

One participant appreciated the quarterly data reviews that allowed clinics to assess and adjust service delivery:

‘Data is essential for planning and improving services. If I was here last quarter and I’m here now, it means I need to do more on this.’ (P11, male, 34 years old)

However, staff shortages occasionally disrupted data review meetings, reducing opportunities for thorough record analysis and feedback:

‘The data review meeting used to be conducted, but due to a lack of staff, we don’t attend all the meetings anymore.’ (P7, female, 39 years old)

### Theme 4: Health financing

A sound health financing system secures adequate funding to ensure that individuals can access necessary health services without risking financial hardship or poverty because of medical expenses.^8^ More than 90% of the participants discussed financial limitations as a recurrent barrier to the provision of care, affecting nearly all facets of CECAP services in both regions.

#### Sub-theme 4.1: Budget constraints

The Namibian government has implemented Universal Health Care, and all health services in public facilities are free of charge. However, there is no dedicated funding for cervical cancer alone. Thus, the CECAP programme relies heavily on NGO financial support and primarily focuses on HIV-positive patients. While this dependence elevated sustainability concerns, participants appreciated the resources NGOs provided, which allowed facilities to maintain continuous services. One participant expressed appreciation for this partnership:

‘NGOs are collaborating with the government, giving us the necessary stock and supporting us.’ (P6, female, 32 years old)

Another participant noted the benefit of this funding, particularly for reaching remote communities:

‘With the support of NGO, we have been able to keep services running and reach out to more women, especially those that live in the inland.’ (P7, female, 39 years old)

However, participants expressed concerns about the absence of a district-specific budget, which made consistent outreach difficult and often drove health workers to resort to informal funding mechanisms such as the use of personal funds and other sales to meet agent needs. One participant emphasised this point:

‘We do not have a budget at the district level, only at the region, and the competition is high. Basically, first come, first serve. So, when there is no money, and we want to buy, for instance, curtains, staff members contribute money. Sometimes, we sell the empty containers from the pharmacy to generate more [*money*].’ (P9, male, 40 years old)

### Theme 5: Access to essential medicines

A functional health system ensures fair access to high-quality, safe, effective and cost-efficient medical products, vaccines and technologies supported by scientifically sound practices.^8^ This theme encompasses the shortages in supplies, equipment and infrastructure necessary for effective CECAP.

#### Sub-theme 5.1: Supplies for screening services

Essential screening supplies were generally available in many clinics, allowing for continued VIA and Pap smear services. However, for early-stage screenings and treatment for positive cases, participants reported adequate resources, although there were no HPV screening services. One participant noted:

‘When a patient presents with precancerous cells, for stage one and two treatments, we manage [*it*] here at the district hospital, there are two trained doctors.’ (P7, female, 39 years old)

However, all participants from one district hospital reported facing recurring shortages of supplies for cervical cancer screening, requiring rescheduling of patients. A participant noted the need for consistent supply management, saying:

‘Pap smear testing kits usually run out of stock, then we rebook the patients, especially those who are above 60 years, and give them a date to come back, but sometimes most of them do not come back and only come back when they are sick.’ (P11, male, 34 years old)

A shortage of educational materials was also highlighted, which hindered awareness efforts, as one participant mentioned:

‘The ministry does not provide pamphlets anymore. Giving health information from the mouth without giving pamphlets is ineffective.’ (P7, female, 39 years old)

#### Sub-theme 5.2: Equipment and infrastructure

Health facilities require improved infrastructure, including updated equipment and sufficient storage, which facilitates the delivery of quality care. Basic amenities such as examination beds often need to be improved, impacting patient comfort and care quality. A participant remarked:

‘We don’t even have examination beds like this one I am using belongs to casualty, and they can come to get it anytime. Sometimes even this lamp you see here is not working; it’s challenging to provide quality care with such limited resources.’ (P4, female, 48 years old)

### Theme 6: Leadership and Governance

Challenges in governance and leadership reflect a lack of cohesive policies and insufficient community engagement in cervical cancer awareness and prevention.^8^

#### Sub-theme 6.1: Prevention policy

While leadership oversight was evident, strategic support was limited; participants felt that logistical and policy support for CECAP required strengthening. One staff member commented:

‘The absence of a dedicated policy makes it hard to establish structured, long-term programmes for cervical cancer.’ (P5, male, 37 years old)

‘We don’t have a specific policy for cervical cancer here; it’s all managed under general health.’ (P3, male, 51 years old)

#### Sub-theme 6.2: Community involvement

Although there is some collaboration with community leaders, it remains occasional, with limited advocacy for cervical cancer awareness, as most initiatives are focused on other diseases such as HIV/AIDS, TB and malaria. Furthermore, cultural stigma discourages many women from participating in screenings, as reproductive health discussions are deemed taboo in the setting. A participant explained:

‘Cultural beliefs and misinformation that your womb will be removed, and many others, discourage many people from seeking screening; we need more local champions to advocate for awareness.’ (P2, female, 35 years old)

Despite these limitations, the involvement of community leaders in health campaigns from one district was appreciated, as it helped build trust and engage the community. Another participant highlighted:

‘Community leaders are involved, and we hold meetings with them here and there to discuss health issues, including cervical cancer.’ (P4, female, 48 years old)

## Discussion

This study found that while CECAP services are available and actively implemented in both regions, issues related to staffing, funding and essential resources present significant barriers to achieving comprehensive service delivery. CECAP services delivery in these regions is structured into primary and secondary levels yet lacks consistency in coverage and effectiveness. Primary prevention efforts, such as awareness education and vaccination against HPV, are hindered by availability, with vaccines predominantly offered only in private facilities at a cost that is not affordable to many. As noted in studies by Al-Amro et al.^27^ Human papillomavirus vaccination coverage is significantly lower in rural areas, particularly in developing settings where public access to vaccines is restricted. Furthermore, secondary prevention through cervical cancer screening using VIA and Pap smear tests was commonly offered in the district hospitals and some clinics, and community health education efforts were utilised to raise awareness. However, coverage was limited by frequent staff shortages and a need for transportation for outreach. This coverage issue, particularly in low-resource settings, is consistently highlighted in similar studies as a primary obstacle to healthcare access.^27,28,29^ This study finds that such constraints in staffing prohibit the establishment of reliable, effective and continuous screening programmes, which may reduce early detection rates and subsequently negatively affect treatment outcomes. Furthermore, cervical cancer screening programmes are mainly facility based, and this limits outreach to remote areas. This finding is similar to other studies, where geographic isolation remains an essential issue for hard-to-reach populations.^30,31,32,33^ The minimal outreach in hard-to-reach areas reflects a missed opportunity to mobilise and educate communities, potentially reducing uptake because of logistical challenges and lack of awareness.

The study highlighted a lack of adequately trained health professionals, particularly in specialised areas such as gynaecology and oncology. Studies by Legasu et al. and Habila et al.^34,35^ emphasise similar workforce constraints in SSA, noting that health personnel often lack specialised training in women’s health, and the availability of trained staff is often inadequate in rural settings. Moreover, patients with advanced diagnoses of cervical cancer are referred to tertiary facilities kilometres away from where they live, and the booking process and turnaround are often lengthy. This dependency on external health facilities could contribute to delays in diagnosis and treatment. Addressing these challenges may require a dual strategy of improving working conditions in rural areas and enhancing training programmes to produce a workforce equipped for rural and specialised care.

Staff retention issues, particularly among young nurses who relocate to urban areas seeking better living and working conditions, further complicated service continuity. For instance, it was identified that only one or two CECAP-trained nurses were available per district, often requiring rotational service to multiple clinics. These findings align with those of Mugassa and Frumence,^30^ who reported that rural-to-urban migration among healthcare workers disrupts rural healthcare service delivery. On the contrary, positive findings emerged, such as the support from health extension workers and community leaders who played active roles in outreach and health education activities.

Moreover, the study revealed that data management was largely manual, limiting the potential for effective data collection, storage and analysis. Although some electronic tools were in place in some facilities, equipment shortages and Internet connectivity issues were reported to hamper their effectiveness. The study further found that even though manual data collection was consistent, users often perceived the process as ineffective because of its inability to be integrated into strategic planning. The limited use of collected data for actionable decisions in these regions could indicate a more significant trend seen in other studies, where data collection in low-resource settings tends to be administrative rather than strategic.^36,37^ The study extends this knowledge by showing that there is a need for targeted training in data analytics and a shift in data culture, where data are viewed not merely as a reporting tool but as a resource for improving patient care.

Additionally, funding was noted to be a critical barrier, highlighting that NGOs primarily funded CECAP services. This is consistent with previous findings highlighting that cervical cancer services in SSA are often donor dependent, with NGO funding accounting for a substantial share of resources.^10,38^ While this support has been invaluable, the limited dedicated government budget for cervical cancer services raises sustainability distress, especially if NGO support were to diminish. Furthermore, it was noted that the current health financing model in Namibia is solely based on a regional budget rather than district-specific allocations, resulting in deferrals and competition for the already inadequate funds. The merged funding approach often limits the agility of district hospitals in addressing immediate needs. Ampofo et al. also observed this challenge in decentralised health systems.^31^ Moreover, the study identified financial constraints that compel staff to spend personal money to meet urgent needs. This pattern highlights an informal mode of financing in resource-scarce health systems. The use of petty cash generated from non-essential sales at health facilities, such as empty pharmacy containers, reflects resourcefulness yet also highlights systemic underfunding. Introducing dedicated district budgets could enhance financial autonomy, as seen in models from other low- and middle-income countries, where decentralised funding enables quicker responses to localised needs.^39^

Additionally, this study found that although some screening equipment is generally available at district hospitals, clinics and outreach points, it often lacks portable screening equipment, such as foldable beds and privacy tents. The need for portable screening equipment is emphasised in similar studies, where limited resources for mobile units directly affect the accessibility of cancer screenings in rural areas.^40,41^ The availability of these resources is essential to expand access to preventive services in geographically isolated areas.

Finally, leadership and governance challenges, especially the double roles of healthcare managers, affect the coordination and delivery of healthcare services, including CECAP. Health managers struggle to balance administrative duties with direct patient care responsibilities and may face time constraints that hinder their involvement in operational oversight. This challenge echoes the findings by Obol et al.,^42^ who argue that effective leadership requires adequate time for both administrative and clinical responsibilities.

### Limitations

The results are contextual and cannot necessarily be extrapolated to other areas, particularly urbanised regions of either Namibia or other nations. Additionally, this is a qualitative study based on the perceptions of participants, which may lead to response bias. However, triangulating the new findings with some quantitative data would allow for a comprehensive description of the challenges of CECAP.

## Conclusion

The cross-cutting challenges to CECAP observed in this study highlight systemic barriers that will need many solutions. Improving access to CECAP services is vital to tackle service delivery issues, workforce shortages and retention issues, health information challenges, financial constraints and pervasive governance gaps. This study adds to the literature by identifying region-specific barriers and potential evidence-based solutions in policy, such as district-specific budgets, improved workforce training and increased access to mobile service screening. These recommendations, if implemented, will enhance the CECAP framework within Namibia and could be a model for other low-resource settings.

Recommendations to policy and practice:

- The Ministry of Health and Social Services (MoHSS) needs to allocate district-specific budgets to support regionally tailored CECAP efforts.
- MoHSS is encouraged to improve training and retention of healthcare workers who are involved in cervical cancer screening and health education services.
- The MoHSS needs to support and expand access to mobile screening services to reach remote and underserved populations.
- Strengthening health information systems is critical to improve tracking, monitoring and reporting of cervical cancer services.
- The MoHSS needs to incorporate community engagement strategies to address cultural norms and improve cervical cancer screening uptake.

Recommendations for future research:

- Evaluate the cost-effectiveness of mobile cervical cancer screening in rural areas.
- Explore culturally sensitive interventions aimed at enhancing community awareness towards cervical cancer screening.
- Investigate the role of digital health technologies in improving health information management and follow-up care in cervical cancer screening programmes.
